# Vestibular characterization in the menstrual cycle

**DOI:** 10.1016/S1808-8694(15)30655-8

**Published:** 2015-10-19

**Authors:** Cintia Ishii, Lucia Kazuko Nishino, Carlos Alberto Herrerias de Campos

**Affiliations:** 1 Master's degree student, speech therapist; 2 Specialist in audiology, Conselho Federal de Fonoaudiologia; speech therapist, Irmandade da Santa Casa de Misericordia de Sao Paulo; 3 Doctorate, adjunct professor, Faculdade de Ciencias Medicas da Santa Casa de Sao Paulo

**Keywords:** hearing, menstrual cycle, electronystagmography, dizziness

## Abstract

Hormonal disorders in the menstrual cycle can affect labyrinthine fluid homeostasis, causing balance and hearing dysfunctions.

**Study Design:**

Clinical prospective.

**Aim:**

compare the results from vestibular tests in young women, in the premenstrual and postmenstrual periods.

**Materials and Methods:**

twenty women were selected with ages ranging from 18 to 35 years, who were not using any kind of contraceptive method for at least six months, and without vestibular or hearing complaints. The test was carried out in each subject before and after the menstrual period, respecting the limit of ten days before or after menstruation.

**Results:**

there was a statistically significant difference in the menstrual cycle phases only in the following vestibular tests: calibration, saccadic movements, PRPD and caloric-induced nystagmus. We also noticed that age; a regular menstrual cycle; hearing loss or dizziness cases in the family; and premenstrual symptoms such as tinnitus, headache, sleep disorders, anxiety, nausea and hyperacusis can interfere in the vestibular test.

**Conclusion:**

there are differences in the vestibular tests of healthy women when comparing their pre and postmenstrual periods.

## INTRODUCTION

Levels of ovarian steroid hormones (estrogen and progesterone) vary according to each phase of the regular menstrual cycle; this variation is controlled by the hypothalamic-hypophyseal-ovarian system.^1^

The menstrual cycle may be divided into the follicular and luteal phases. The follicular phase (or early follicular or menstrual phase) is characterized by low estrogen and progesterone levels. In the luteal phase (or late luteal or pre-menstrual phase), estrogen and progesterone levels are decreased.^2^

Most of the changes in women take place in the luteal phase; these changes include fluid retention, weight gain, increased energy demands, changes in glucose uptake, a slower gastrointestinal transit time, altered lipid profiles, altered vitamin D, calcium, magnesium and iron metabolism, emotional hypersensitivity, generalized pain, and changes in dietary habits.^3^ Other findings in this phase are hydrops of the labyrinth (due to sodium retention and the resulting endolymphatic hypertension) noise intolerance, and an “empty head” feeling.^4^

Hormone alterations in the menstrual cycle, pregnancy and menopause may compromise the homeostasis of labyrinthic fluids, since they act directly on enzymatic processes and in neurotransmitter effects; these changes may alter balance or hearing.^5^

A study of premenstrual dizziness has suggested that peripheral vestibular alterations may occur due to fluid retention in the luteal phase of the ovarian cycle^6^ resulting from increased estrogen, progesterone and aldosterone release.^7^ Other findings may be vertigo or dizziness a few days before menstruation, due to increased levels of estrogen, progesterone and aldosterone in the inner ear. The effect of such increased hormone levels is hydrops of the labyrinth and symptoms similar to those encountered in Ménière's disease.^7^

Because of this possible influence of ovarian hormones in different phases of the menstrual cycle on the vestibular function, there has been interest in seeking vestibular signs that might demonstrate such effects.

Thus, the purpose of this study was to verify whether vestibular examination results differed in women during the pre- and postmenstrual period.

## METHOD

All participants were informed about the aims of this study and were invited to take part after signing a free informed consent form. This study complied with the principles of ethics in research on human beings contained in the resolution 196/96 (Ministry of Health, 1996) and the guidelines of the Research Ethics Committee (protocol 02/06).

Twenty females aged from 18 to 35 years were selected for this study.

Inclusion criteria were females that:

1) had not used any hormonal contraceptives within the last 6 months;
2) had audibility thresholds from 0.25 kHz to 8 kHz, speech recognition threshold of up to 25 dBHL, and speech recognition of monosyllables of at least 88%.
3) had intact external acoustic meatuses (demonstrated by meatoscopy);
4) had no vestibular complaints, verified by a questionnaire consisting of: identification, otological history, auditory complaints, vestibular complaints, and information about the menstrual cycle.

After confirming the inclusion criteria, vestibular testing was done as follows: Positional Nystagmus Test (PN), Calibration (CAL), Spontaneous Nystagmus Test with Open Eyes (SNOE) and with Close Eyes (SNCE), Semi-spontaneous Nystagmus with Open Eyes (SSNOE), Saccadic Movements (SMs), Pendular Tracking (PT), Optokinetic Nystagmus Test (ON), Damped Pendular Rotating Test (DPRT), and Air Caloric Test (CT) at 42°C and 18°C.

Each participant was asked not to consume chocolate, coffee, black tea, mate, green tea, and alcoholic beverages, and to avoid smoking during the three days before testing.

All tests were recorded in a computer; automatic calculation of gain, latency, precision, and angular velocity of the slow component of nystagmus and all other necessary calculations for each test.

The computer of the digital vectoelectronystagmography device includes a specific software, a lighted bar and an air otocalorimeter (type NGR 07 - Neurograff Eletromedicina Ind. & Com. Ltda).

Audiological testing was done in an acoustically treated booth. An Interacoustics model AC 40 audiometer and TDH 39P Telephonics headphones were used. Acoustic immittance measurements were tested using an Interacoustics model AZ7R immittance testing device.

A 5% significance level was adopted for statistical purposes. The SPSS (Statistical Package for Social Sciences) software, version 13.0, was used for applying Wilcoxon's signed rank test and Spearman's correlation analysis.

## RESULTS

The results of vestibular testing were within normal limits.

**Figure 1:**
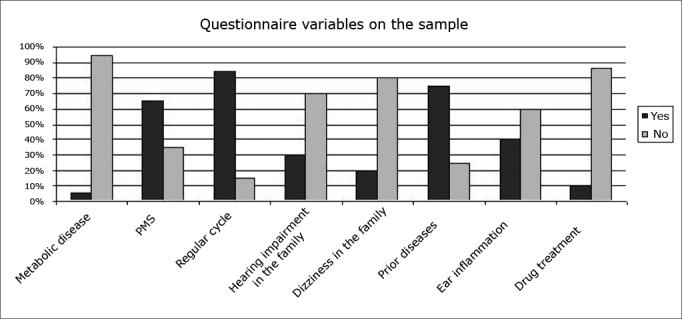
Percentage of occurrence of questionnaire variables in the sample.

**Figure 2:**
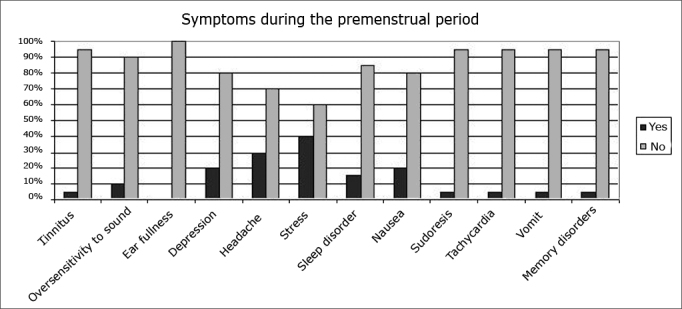
Percentage of symptoms occurring during the premenstrual period in the sample.

The questionnaire revealed the main variables for characterizing the sample and the symptoms occurring across the menstrual phase of the cycle (Figure 1, Figure 2).

Wilcoxon's signed rank test demonstrated possible differences between the two points we took into account (Table 1). There were statistically significant differences among the phases of the menstrual cycle in the following tests: CAL latency in the right eye, SM precision in the left eye, DPRT (superior canal) directional preponderance (DPN) of nystagmus, and in CAL, slow component angular velocity (SCAV) at 42°C.

**Table 1:** Comparison of results of menstrual cycle phases in vestibular testing.

Pair of Variables	Mean	Standard deviation	Significance (p)
CAL Latency RE pre	179,54	56,70	0,042 *
CAL Latency RE post	148,99	35,54	
SM Precision LE pre	0,98	0,11	0,048 *
SM Precision LE post	1,06	0,19	
DPRT DPNS pre	0,11	0,06	0,017 *
DPRT DPNS post	0,15	0,08	
CT 42° SCAV RE pre	5,86	1,75	0,050 *
CT 42° SCAV RE post	7,12	2,89	

Key:

RE = Right ear

LE = Left ear

CAL = calibration

SMs = Saccadic movements

DPRT = Damped pendular rotation test

DPNS = Directional preponderance of nystagmus of the superior canal

CT = Caloric test

SCAV = Slow component angular velocity

* significant p-values in Wilcoxon's signed rank test.

Spearman's correlation analysis showed the relation among questionnaire variables and vestibular test results.

Table 2, Table 3 describe the significant p-values among the questionnaire variables and the symptoms in the menstrual phase, and vestibular tests.

**Table 2:** Result of statistically significant p-values in vestibular tests and questionnaire variables, for the pre- and postmenstrual phases.

Pre-menstrual	Duration of cycle	Deafness in family	Dizziness in family	Previous disease	Treatment with medication
CAL Velocity				LE = 0,006	
SM Latency				LE = 0,019	
PT Type		0,002			
ON DPN				0,008	0,023
DPRT Velocity PSC	LE = 0,034				
DPRT DPNS			0,025		
Postmenstrual	Age	Regulated cycle	Deafness in family	Previous disease	Treatment with medication
CAL Latency	LE = 0,004				
CAL Velocity		LE = 0,035		RE = 0,028	
SM Latency					RE = 0,035
PT 0.40 Hz			0,027		
ON DPN			0,006		0,019

Key:

RE = Right ear

LE = Left ear

CAL = calibration

SMs = Saccadic movements

PT = Pendular tracking

ON = Optokinetic nystagmus

DPN = Directional preponderance of nystagmus

DPRT = Damped pendular rotation test

DPNS = Directional preponderance of nystagmus of the superior canal

PSC = Posterior semicircular canal

**Table 3:** Result of statistically significant p-values in vestibular tests and pre- and postmenstrual cycle symptoms

Premenstrual	Tinnitus	Headache	Sleep disorder	Anxiety	Nausea
PT Type	0,013				
PT 0.20 Hz		0,016			
PT 0.40 Hz		0,001			
ON SCAV		RE = 0,049			
ON Gain			RE = 0,040		
DPRT Velocity LSC					RE = 0,038
DPRT Velocity PSC		LE = 0,044		RE = 0,026	LE = 0,010
DPRT Velocity SSC		RE = 0,049		LE = 0,012	RE = 0,038
CT SCAV 18°		LE = 0,028			
Post-menstrual	Hypersensitivity to loud sounds	Headache	Anxiety	Sleep disorder	Nauseas
CAL Precision				RE = 0,009	
SM Precision	RE = 0,040			RE = 0,013	
				LE = 0,009	
PT 0.10 Hz				0,013	
PT 0.20 Hz		0,035		0,013	
PT 0.40 Hz				0,004	
ON SCAV			RE = 0,026	RE = 0,018	
			LE = 0,027	LE = 0,013	
ON Gain				RE= 0,012	
DPRT Velocity PSC				RE = 0,026	
				LE = 0,013	
DPRT Velocity SSC				RE = 0,026	
				LE = 0,013	
DPRT DPNL			0,021		
DPRT DPNS	0,040		0,003		0,002
CT SCAV 42°			LE = 0,029	RE = 0,004	
				LE = 0,030	
CT SCAV 18°		RE = 0,021	RE = 0,023	RE = 0,013	
				LE = 0,006	

Key:

RE = Right ear

LE = Left ear

CAL = calibration

PT = Pendular tracking

ON = Optokinetic nystagmus

SCAV = Slow component angular velocity

DPN = Directional preponderance of nystagmus

DPRT = Damped pendular rotation test

DPNL = Directional preponderance of nystagmus do canal lateral

DPNS = Directional preponderance of nystagmus of the superior canal

LSC = Lateral semicircular canal

PSC = Posterior semicircular canal

SSC = Superior semicircular canal

SM = Saccadic movements

CT = Caloric test

## DISCUSSION

The menstrual cycle may be defined as the interval between the first day of a menstruation and the first day of the next menstruation. Premenstrual tension (PMT) is a set of symptoms that arise between 10 and 14 days before menstruation and disappears after it begins. Over 150 symptoms have been catalogued; their incidence varies and is not constant. When these symptoms become intense to the point of interfering with daily activities, they compose the severe form of PMT, the Premenstrual Syndrome (PMS). PMT affects about 75% of women in the reproductive age.^8^, ^9^, ^10^

A decreased CAL latency and an increased SM precision during the postmenstrual period diverge from the findings of a study^11^ that monitored two menstrual cycles of 12 subjects and concluded that hormone changes within the cycle had no significant effect on the optokinetic function, and that only lateral postural stability was affected.

It is important to note that estrogen and progesterone levels in the premenstrual phase may affect central nervous system functioning, indirectly altering the optokinetic function. This may occur especially in those areas related to the visual-vestibular interaction, such as GABAA (gamma-aminobutyric acid) receptors, which is an inhibitory neurotransmitter that binds to specific receptors. Progesterone metabolism may modulate these receptors, altering the transmission in the vestibular nuclei that are involved with the optokinetic, vestibuloocular and vestibulospinal reflexes.^11^

Increased DPN values in the upper semicircular canals in the DPRT test during the postmenstrual phase were statistically significant. We may assume that this occurred due to a possible effect of sex hormones on bodily fluids.^12^ Thus, as the volume and pressure of endolymph and perilymph increased during the premenstrual phase, stimulation during the test would have a milder effect when we positioned the head for the DPRT of the vertical canals to the right and left.

In the CT at 42°C, an increased postmenstrual SCAV value was also statistically significant. We may assume that this occurred in the premenstrual period because the baseline temperature of the female body increases as a results of increased sexual hormone levels.^13^ Thus, the effect on the labyrinth would be dampened when stimulating the ear at 42°C in the premenstrual period, compared to the postmenstrual period.

Crossing the questionnaire variables and the symptoms demonstrated in the results of vestibular tests during the premenstrual phase allowed us to raise some hypotheses about such correlations.

Age may affect CAL latency due to the natural reduction of bodily muscle movements and degeneration of hair cells, otoliths, ganglion cells and nerve endings in the peripheral and central vestibular system, all of which may be observed in the elderly.^14^ The menstrual cycle being regulated or not may affect the CAL velocity; the more regulated the cycle, the more regulated will be the sex hormone levels, which would cause fewer changes in the inner ear.

Cases of deafness and dizziness in the family may suggest a genetic predisposition to develop labyrinthic and cochlear alterations;^15^ which could also affect the vestibular tests. A history of diseases such as measles and chickenpox, and treatment with certain mediations may affect the results of the CAL velocity and latency, SM latency, PT type and frequency and DPN, ON DPN, and DPRT velocity and DPN, since these are risk factors for the presence of inner ear alterations. Viral diseases may cause endolymphatic labyrinthitis or neuritis of the VIII cranial nerve, which may cause sensorineural hearing loss. Otitis media and medication therapy may injure the base of cochlea.^15^

Tinnitus, headaches, sleep disorders, anxiety, nausea, and hypersensitivity to sound may alter the vestibular tests (PT type and frequency, ON gain and SCAV, DPRT velocity and DPN, and CT SCAV), since the premenstrual period alters perilymph and endolymph pressure and blood viscosity.^12^ Additionally, some studies have shown that there are psychic symptoms in the PMS that decrease the concentration ability,16 which also reduces attention during testing.

Female sex hormones thus alter the vestibule physically - changing the endolymphatic pressure - and blood viscosity. The most significant changes, however, are those that result from the effects of progesterone and estrogen on the central nervous system (neurotransmitters and their interactions).

## CONCLUSION

Based on these data we concluded that:

In different phases of the menstrual cycle the following tests are altered: CAL, SMs, DPRT and CT SCAV at 42°C.
